# Development of Octyl Methoxy Cinnamates (OMC)/Silicon Dioxide (SiO_2_) Nanoparticles by Sol-Gel Emulsion Method

**DOI:** 10.3390/nano7120434

**Published:** 2017-12-07

**Authors:** Pey-Shiuan Wu, Yi-Ching Lee, Yi-Ching Kuo, Chih-Chien Lin

**Affiliations:** Department of Cosmetic Science, Providence University, Taichung 43301, Taiwan; jwu2@pu.edu.tw (P.-S.W.); g1040050@pu.edu.tw (Y.-C.L.); sin14990@gmail.com (Y.-C.K.)

**Keywords:** octyl methoxy cinnamates (OMC), OMC/SiO_2_ nanoparticles, sol-gel emulsion method, tetraethoxy silane (TEOS), in vitro release profile

## Abstract

Although octyl methoxy cinnamates (OMC) is the most used Ultraviolet B (UVB) filter in sunscreen, it has poor light stability in emulsion system. In this study, OMC/SiO_2_ nanoparticles were prepared via sol-gel emulsion method. Tetraethoxy silane (TEOS) was used as the silica source to encapsulate OMC. Modification of experimental parameters such as stirring speed of condensation reaction and emulsion condition, pH value of acid-catalyzed, surfactant and different percentage of TEOS and OMC, adding of OMC and surfactant to different phase may affect the particle size, and yield and entrapment efficiency in preparation process of OMC/SiO_2_ nanoparticles. Concluding all the parameter, we found that when condensation reaction and emulsion conditions are at 1000 rpm, pH 1.5, Span 80/Tween 20, TEOS/OMC ratios 1:1, OMC and surfactants added in oil phase, resulting in smaller particle sizes 476.5 nm, higher yield 95.8%, and higher entrapment efficiency 61.09%. Fourier transform infrared (FTIR) analysis demonstrated that OMC/SiO_2_ nanoparticles were successfully prepared. In vitro release profile supposed that OMC/SiO_2_ nanoparticles can delay OMC releasing and had 60.83% decreasing of cumulative amount. Therefore, the OMC/SiO_2_ nanoparticles have the potential to develop as new sunscreen materials in the use for cosmetics field in the future.

## 1. Introduction

Ultraviolet (UV) radiation is separated based on different wavelength into three categories Ultraviolet C (UVC) 280–100 nm, Ultraviolet B (UVB) 315–280 nm and Ultraviolet A (UVA) 400–315 nm, respectively. Regarding the ultraviolet radiation that reaches the Earth’s surface, about 95% is UVA and 5% is UVB. The ozone layer absorbs 97–99% of UV irradiation [1]. In recent years, industrial development has caused serious destruction of the ozone layer; the probability of human body exposure to UV radiation increases [2]. Exposure to UV irradiation has been confirmed to have harmful effects on the skin such as damage, and acceleration of the aging process in human skin. In literature, UV irradiation has been shown to impair collagen type I synthesis and decrease collagen production [3]. Collagen, elastin, and fibrillar structure in photoaged skin is incomplete if compared to a sun-protected chronologically aged skin. Photoaged skin appears lax, wrinkled, with uneven pigmentation and coarse, brown spots and cause skin and pancreatic cancer easily [4,5]. Consequently, sunscreens have become important for the protection of skin [6].

Octyl methoxy cinnamates (OMC) is one of the most used UVB filters in sunscreens. It is commonly used to absorb from 280 to 310 nm [7]. Although OMC is often used with UVA absorbers to achieve Broad Spectrum in order to protect skin and prevent sun exposure, it has poor photostability in emulsion system. Furthermore, half of the activity will photodegrade via 10 minimal erythema dose (MED) UV radiation, thus leading to decreased sun protection [8]. In another study, photodegraded products of OMC may lead to allergic reactions or cause dermatitis [9]. Some experiments showed that OMC possesses estrogenic activity in animal models; groups of 14 to 18 pregnant Wistar rats were dosed with 0, 500, 750, or 1000 mg OMC/kg bw/day during gestation and lactation. On postnatal day 16, high-dose male offspring showed reduced relative prostate and testis weights and a dose-dependent decrease in testosterone levels and motor activity levels [10]. Consequently, many researches focused on UV filters encapsulation to avoid high concentrations that absorbed into skin and light degradation products causing skin damage [11,12,13].

Mesoporous silica nanoparticles have been broadly investigated in recent years because their wide range of potential applications, due to the outstanding properties including large pore volumes, tunable pore sizes, high surface areas, and good biocompatibility [14], which are highly attractive in technical sciences such as catalysis, electronics, and photocatalytic hydrogen production [15]. Compared with mesoporous silica nanoparticles, hollow silica nanoparticles exhibit unique advantages with huge cavities and mesoporous shells and show excellent performance in many fields such as air or water purification, catalysts immobilization, drug delivery, and sunscreen development [16,17]. However, no earlier study has encapsulated the OMC molecules into silica nanoparticles. Therefore, in the presented study, we supposed that the silica encapsulated OMC nanoparticles (OMC/SiO_2_ nanoparticles) may have a chance to obtain better properties for photostability and phototoxicity.

The hard template uses colloidal particles to prepare hollow silica nanoparticles, including mesoscale silica spheres, polystyrene (PS) beads, and nanoparticles of various materials such as carbon, metals, and metal oxides. The template surface was modified in order to achieve favorable surface properties. Selective method was used to remove templates in order to obtain the hollow structures [18]. However, the process of preparation is complicated, and its high manufacturing costs make it scarce in the market. Compared to hard template, soft templates have their own advantages such as simple procedures and variety of the template sources (such as vesicles, liquid droplets, and emulsion droplets). Hollow silica nanoparticles via self-assembly of surfactants or macromolecule polymer as a structure-directing agent to prepare [19].

The sol-gel emulsion method uses the emulsion droplets as templates to prepare silica nanoparticles by hydrolysis of tetraethyl orthosilicate (TEOS) [17,20]. The uniformity of particle size and the shell thickness of hollow silica spheres is worse than the hard template method because emulsion droplets are mobile. Therefore, the addition of co-solvent such as ethanol to stabilize the particles can prevent agglomerations [21]. Although the sol-gel emulsion method and silicon dioxide are commonly used method and substance for the preparation of nano-scale particles, there is no study that incorporates the UV filter OMC into silicon dioxide nanoparticles. Therefore, in this study, the nanoparticle used TEOS as the silica source to encapsulate OMC prepared by the sol-gel emulsion method. The OMC was encapsulated to avoid light degradation or prevent phototoxicity and contact dermatitis. Consequently, OMC/SiO_2_ nanoparticles may have chance to be used as a potent and safe sunscreens for the cosmetic industry in the future.

## 2. Results and Discussion

### 2.1. Characteristics of OMC/SiO_2_ Nanoparticles

In the presented study, the experimental parameters are shown in Table 1. To develop the suitable OMC/SiO_2_ nanoparticles, we tried to find the best conditions for the production of the OMC/SiO_2_ nanoparticle. In addition, the conditions of OMC and surfactant added in different phases are shown in Table 2.

**Figure 1:**
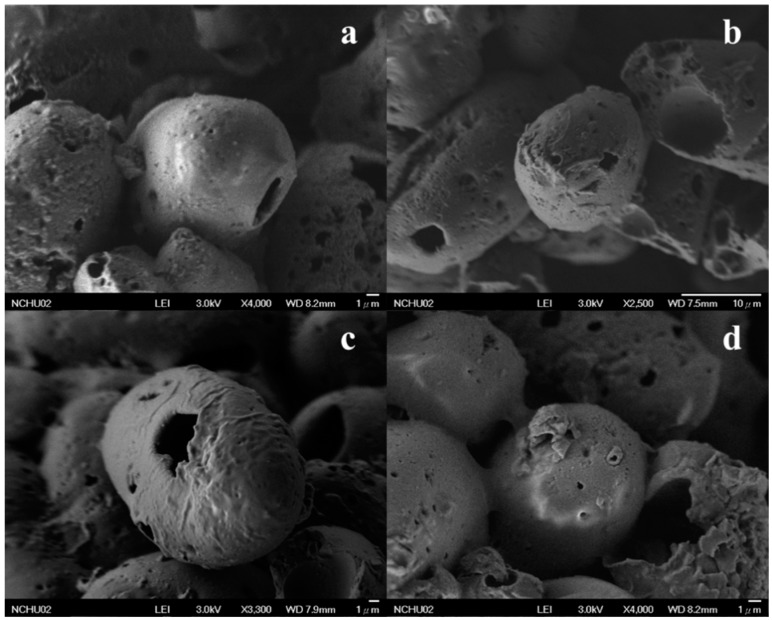
The scanning electron microscopy (SEM) images of the OMC/SiO_2_ nanoparticles (**a**,**b**) and hollow spheres of OMC/SiO_2_ nanoparticles (**c**,**d**).

In the presented study, we synthesized the OMC/SiO_2_ particles through the encapsulation of UV filter OMC into the core of SiO_2_ spheres, not by the attraction of surface area of particles. The scanning electron microscopy (SEM) images of OMC/SiO_2_ nanoparticles (Figure 1a,b) indicated that the nanoparticles of spheres had a rough surface and remained spherical in morphology. The SEM image (Figure 1c,d) also showed that the spheres were hollow. However, although the used surfactants in OMC/SiO_2_ particles surface are not removed by the calcination process, there are still formed by cluster of numerous silica nanoparticles with pores on the surface.

### 2.2. Particle Sizes and Yield of OMC/SiO_2_ Nanoparticles

The results of particle sizes, polydispersity index (PDI), and yield of OMC/SiO_2_ nanoparticles are presented in Table 3. Sample 1 shows that stirring speed had a relatively large particle size of OMC/SiO_2_ nanoparticles (1163.3 nm). With speeds up to 1000 rpm, the stirring speed had a relatively narrow particle size (1093.2 nm), and the highest yield was 79.9%. The result shows that the stirring speed of emulsification will affect the size of the emulsion micelles caused by varying particle size.

Samples 3, 4, and 5 showed that the particle size is reduced as the pH value decreased. When the pH value was 1.0, a relatively narrow particle size of 900 nm was detected. However, when the pH value was 1.5, a similar particle size (1093.2 nm) and higher yield (79.9%) were detected. Since the hydrolysis reaction was faster than the condensation reaction, several silicon monomers were formed, thus causing smaller particles and higher yield. Therefore, pH value at 1.5 was suggested as one of the best production condition.

Samples 6 and 7 revealed that water-in-oil (W/O) emulsion system lead to larger particle size 1093.2 nm when Span 80 was used in the emulsion medium. On the contrary, the addition of Tween 20 to the system forms the O/W emulsion system with smaller emulsion droplets, which resulted to the smaller particle sizes (683 nm) and higher yield (87.19%). In Sample 7, TEOS/OMC at 1:1 had smaller particle size of OMC/SiO_2_ nanoparticles (683 nm) and higher yield (87.19%). When the internal phase of emulsion droplets increased, the concentration of the ingredient in the particle’s core increased; this caused a large particle size (1237 nm) when TEOS/OMC was 1:2. The lower PDI at TEOS/OMC was 1:2 due to the similar ratio of aqueous and oil; this caused uniform emulsion droplets and had better PDI.

According to the above results, the best production conditions are the condensation reaction and emulsion condition at 1000 rpm, pH 1.5, Span 80/Tween 20, and TEOS/OMC at 1:1, with smaller particle sizes (683 nm) and higher yield (87.19%). Samples 7, 10, and 11 show the influence of particle size and yield of OMC/SiO_2_ nanoparticle, if OMC and surfactant were added in different phase. The result shows that when OMC and surfactant were added in oil phase, a smaller particle size (476.5 nm) and higher yield (95.8%) were formed. All the PDI of samples were lower than 0.7, and the result indicated that all the particle size distribution of the samples were moderate dispersion system.

### 2.3. Entrapment Efficiency (EE) and Loading Capacity (LC) of OMC

**Figure 2:**
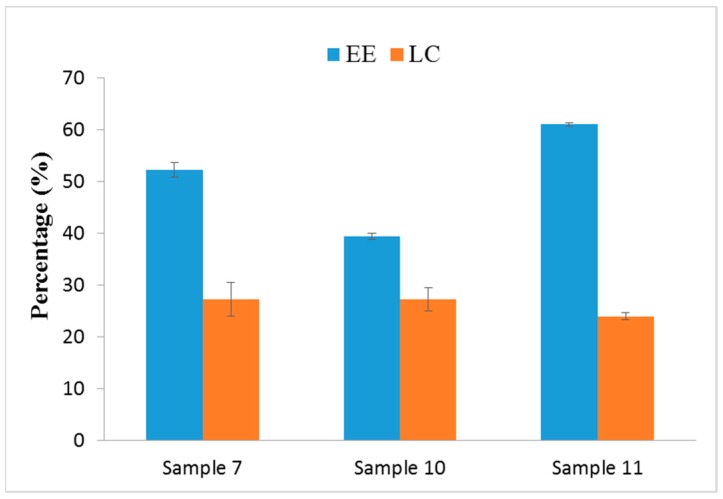
Entrapment efficiency (EE) and loading capacity (LC) of OMC/SiO_2_ nanoparticles.

For the entrapment efficiency (EE) and loading capacity (LC) studies, the EE of Samples 7, 10, and 11 was 52.26%, 39.46%, and 61.09%, respectively. The LC of Samples 7, 10, and 11 was 27.26%, 27.32%, and 24%, respectively (Figure 2). The result shows that sample 10 had lower EE due to the addition of OMC and surfactant in water phase, which caused the instability of the emulsion system and lead to incomplete entrapment.

Sample 11 was shown to be a dispersion system, which leads to smaller micelles when oil soluble of OMC and surfactant homogeneous were mixed in the oil phase. This enhances the adsorption of OMC to nanoparticles and had higher EE (61.09%). However, higher yield and the narrow space of the smaller particle size occasioned lower LC.

### 2.4. Fourier Transform Infrared (FTIR) Analysis of OMC/SiO_2_ Nanoparticles

**Figure 3:**
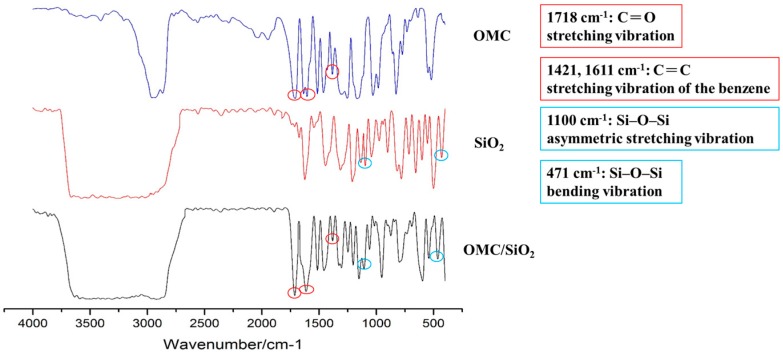
Fourier transform infrared (FTIR) spectroscopy of OMC/SiO_2_ nanoparticles.

FTIR spectra for OMC, SiO_2_, and OMC/SiO_2_ nanoparticles were carried out to confirm the compositions of the prepared OMC/SiO_2_ nanoparticles. The major characteristic bands of OMC were at 1718 cm^−1^ for C = O stretching vibration and at 1421, 1611 cm^−1^ for C = C stretching vibration of the benzene. Furthermore, the characteristic bands of SiO_2_ nanoparticles at 1100 cm^−1^ is associated with the Si–O–Si asymmetric stretching vibration and at 471 cm^−1^ is associated with the bending vibration of Si–O–Si. In comparison, the infrared (IR) spectrum of OMC/SiO_2_ nanoparticles also presents these characteristic bands (Figure 3). Therefore, these results confirmed that OMC were successfully combined with SiO_2_ nanoparticles [22].

### 2.5. In Vitro Release Profile

**Figure 4:**
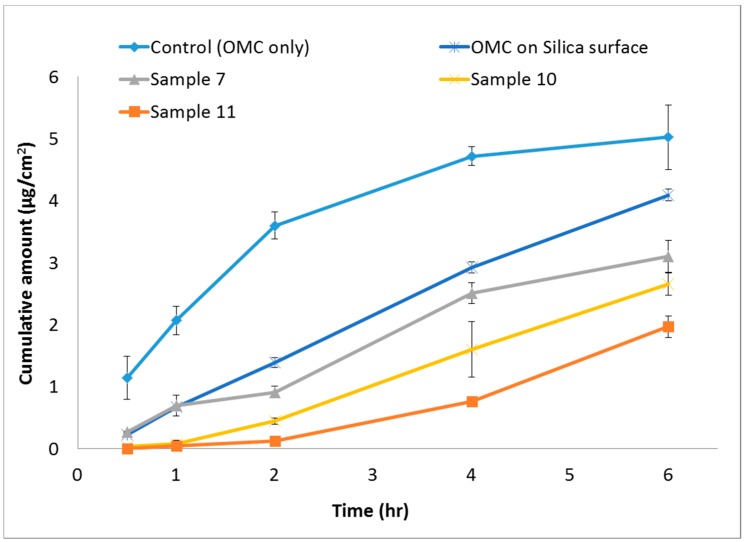
Release profile of OMC from OMC/SiO_2_ nanoparticles.

The release properties of OMC in vitro were shown in Figure 4. The OMC of Samples 7, 10, and 11 released from nanoparticles were obviously slower than that of control (OMC only) and OMC on silica surface groups. In 2 h, 3.60 ± 0.22 μg/cm^2^ of OMC was released, whereas Samples 7, 10, and 11 only released 0.91 ± 0.10, 0.45 ± 0.05, and 0.13 ± 0.03 μg/cm^2^, respectively. Control showed the highest cumulative released 5.03 ± 0.52 μg/cm^2^ at 6 h. In contrast, only 3.11 ± 0.26, 2.65 ± 0.18, and 1.97 ± 0.17 μg/cm^2^ of OMC were cumulatively released from the Samples 7, 10, and 11. Besides, for OMC on silica surface group, the cumulative amounts of OMC were all higher than that of OMC/SiO_2_ particles groups from 2 to 6 h. There is one previous study that used the prepared silica particles that only absorb OMC on the surface to improve OMC safety and photostability [23]. Hence, our results also supposed that the OMC molecules were successfully encapsulated into the SiO_2_ nanoparticles and not only absorbed on the surface.

Compared with three samples, Sample 7 had relatively high cumulative released of OMC. This may be due to the addition of OMC and surfactant in different phase, which cause unstable nanoparticles. In contrast, Sample 11 had the lowest accumulative releasing of OMC, and compare with control had 60.83% of cumulative released decreasing; it may form more stable nanoparticles than Sample 7 and 10 nanoparticles. OMC with surfactant all added in oil phase lead to dispersion system and formed regular and stable micelles. The result confirmed that OMC/SiO_2_ nanoparticles could delay the OMC releasing. This property may protect OMC, avoid light degradation or phototoxicity, and also prevent dermatitis.

The inorganic UV filter such as TiO_2_ has a few good properties for use in sunscreen. However, most sunscreen products on the market are designed by combining organic and inorganic UV filters together in formulation to achieve a high sun protection factor (SPF) or for a broad spectrum purpose. The inorganic UV filters have to be used at a high concentration when not combined with organic UV filter in products. For example, in a lotion type, SPF 30 sunscreen product, single UV filter TiO_2_ content higher than 10 to 15% is essential. In addition, high TiO_2_ content may also increase the instability of formulation. In contrast, organic UV filters often have good UV absorption ability but less photostability. Therefore, in the presented study, the organic UV filter OMC encapsulated into inorganic SiO_2_ particles, which is safer than an original OMC and may reveal the great UV absorption and reflection functions when used with a relative lower content in products. Supposedly, the developed OMC/SiO_2_ particles have the potentials to become a good UV filters for sun protection purpose.

## 3. Materials and Methods

### 3.1. Materials

Tetraethyl orthosilicate (TEOS, >98%) purchased from SHOWA (Tokyo, Japan). Octyl methoxycinnamate (OMC) was purchased from DSM-Pentapharm (Basel, Switzerland). Ethanol and isopropanol was purchased from ECHO CHEMICAL (Miaoli, Taiwan). Nitric acid purchased from SIGMA-ALDRICH (Steinheim, Germany). Sorbitan oleate (Span 80) was purchased from CRODA (Yorkshire, UK). Mineral oil was purchased from TOP RHYME (Taipei, Taiwan). Polysorbate 20 (Tween 20) and Sodium Lauryl Ether Sulphate (SLES) were purchased from Kao (Tokyo, Japan). Methanol was purchased from Alfa Aesar (Ward Hill, MA, USA).

### 3.2. Preparation of the OMC/SiO_2_ Nanoparticles

#### 3.2.1. Hydrolysis and Condensation Reaction

Thirty (30) mL of ethanol were mixed with 10 g of TEOS and 40 mL of distilled water. The solution was acid-catalyzed hydrolysis by nitric acid to pH 1.5 and then dispersed by an ultrasonic bath (DC 300H, DELTA^®^, DELTA Ultrasonic, New Taipei City, Taiwan) for 1 h. TEOS sol was obtained via condensation reaction by heating, then 10 g of OMC was added to the solution and stirred at 1000 rpm for about 40 min at 80 °C.

#### 3.2.2. Emulsion Polymerization

Here, 38.7 g of mineral oil were mixed with 5 g of Span 80 and 5 g of Tween 20. Then, TEOS sol was added and stirred at 1000 rpm for 40 min at 80 °C. OMC/TEOS gel was obtained. Afterward, the samples were collected by centrifugation and washed with ethanol several times. Then, samples were dried overnight at 50 °C to obtain the OMC/SiO_2_ nanoparticles. The change in the experimental parameters is shown in Table 1. We chose the best conditions and discussed the influences to prepared OMC/SiO_2_ nanoparticle. OMC and surfactant added in different phases is shown in Table 2.

### 3.3. Scanning Electron Microscopy (SEM)

The morphology of OMC/SiO_2_ nanoparticles was examined using a field emission scanning electron microscope (FE-SEM, JSM-6700F, JEOL Ltd., Tokyo, Japan). Prior to examination, a small amount of OMC/SiO_2_ nanoparticles were placed onto the carbon-coated copper grids, and a thin layer of gold was sputtered under vacuum onto the samples.

### 3.4. Particle Sizes of OMC/SiO_2_ Nanoparticles

Dynamic light scattering (DLS) is one of the standard methods for measuring particle sizes in fluids. This method is based on the examination of random particle movement due to constant Brownian motion [24]. Prior to examination, 0.05 g of OMC/SiO_2_ nanoparticles added in 10% SLES solution, 1 mL of OMC/SiO_2_ solution was added in quartz tube. Particle sizes and PDI value were assay by Dynamic Light Scattering Nanoparticle Size Analyzer (Malvern Zetasizer Nano ZS, Malvern Instruments Ltd., Malvern, UK).

### 3.5. Yield of OMC/SiO_2_ Nanoparticles

The Yield of OMC/SiO_2_ nanoparticles were calculated using the following equations [25]:

$$\mathrm{Yield}\left(\%\right)=\frac{\mathrm{total}\;\mathrm{amount}\;\mathrm{of}\;\mathrm{nanospheres}}{\mathrm{TEOS}\;\mathrm{amount}+\mathrm{OMC}\;\mathrm{amount}\;}\times 100$$

### 3.6. Entrapment Efficiency (EE) and Loading Capacity (LE) of OMC

The supernatant and the washing solutions were collected together and diluted with ethanol about 1:4 ratio to determine the concentrations of OMC by high-performance liquid chromatography (Agilent HP-1200, Agilent, Santa Clara, CA, USA) with UV-vis (Agilent UV-1575, Agilent, Santa Clara, CA, USA). OMC separation was carried out on a C18 column (Kinetex 5u EVO C18 100A 250 × 4.6 mm, Phenomenex Inc, Torrance, CA, USA) using a mobile phase consisting of methanol-water (80:20, *v*/*v*) at a flow rate of 1.0 mL/min. The detection wavelength was set at 310 nm. The sample was passed through a 0.45 μm polytetrafluoroethylene (PTFE) membrane filter. The OMC entrapment efficiency (EE) and loading capacity (LC) were calculated using the following equations [25]:

$$\mathrm{EE}\;\left(\%\right)=\frac{\mathrm{total}\;\mathrm{OMC}\;\mathrm{amount}-\mathrm{free}\;\mathrm{OMC}\;\mathrm{amount}}{\mathrm{total}\;\mathrm{OMC}\;\mathrm{amount}\;}\times 100$$

$$\mathrm{LC}\;\left(\%\right)=\frac{\mathrm{total}\;\mathrm{OMC}\;\mathrm{amount}-\mathrm{free}\;\mathrm{OMC}\;\mathrm{amount}}{\mathrm{total}\;\mathrm{nanospheres}\;\mathrm{weight}\;}\times 100$$

### 3.7. Fourier Transform Infrared (FTIR) Analysis

FTIR characterization of the samples was carried out using an FTIR spectrometer (FTIR Spectrometer-4100, JASCO, Tokyo, Japan) to scan over a spectral region of 400–4000 cm^−1^ on a thin slice sample, which was compressed from the dry mixture of sample and KBr.

### 3.8. In Vitro Release Profile

The release of OMC/SiO_2_ nanoparticles were by Franz Type diffusion cell (LOGAN FDC-6, LOGAN Instruments, Somerset, NJ, USA); 1.0 mL SiO_2_-OMC emulsion (10 g) was placed in the donor site, and 5.0 mL consisting of phosphate buffered saline (PBS) and ethanol (70:30, *v*/*v*) was poured into the receptor site. These two chambers were separated by a 0.785 cm^2^ artificial skin placed over the aperture of Franz diffusion apparatus. The Franz Type diffusion cell were stirred at 36.7 ± 0.3 °C with magnetic stirrers. Aliquots of 1 mL were withdrawn at intervals and replaced by same volume of fresh medium. The amount of released OMC was passed through a 0.45 μm polytetrafluoroethylene (PTFE) membrane filter, and the amount of released OMC was measured by a high-performance liquid chromatography. For the control (OMC only) group, 24% (*w*/*w*) OMC was directly dissolved in PBS with 30% ethanol (the same amount with the lowest OMC/SiO_2_ sample). For OMC on silica surface groups, 24% (*w*/*w*) OMC was added with the pre-prepared empty silica particles in isopropanol solution and then dried with a rotary evaporator (EYELA, Tokyo, Japan).

### 3.9. Statistical Analysis

The data from experiments were analysed by the Student’s *t*-tests. All of the results are presented by way of means ± S.E. from three independent experiments.

## 4. Conclusions

In this study, we have successfully prepared OMC/SiO_2_ nanoparticles via the sol–gel emulsion method. We used TEOS as the shell material: surfactant, mineral oil, and OMC as oil phase. Water phase is composed of TEOS, ethanol, and water in the ratio of 1:3:4. After acid-catalyzed hydrolysis, TEOS gel was mixed with the oil phase after condensation reaction to form OMC/SiO_2_ nanoparticles. The OMC/SiO_2_ nanoparticles were spherical in morphology and can control the sol-gel conditions or the pattern of emulsification to adjusted particle size or yield.

We found that the best production condition was the condensation reaction and an emulsion condition at 1000 rpm, pH at 1.5, Span 80/Tween 20, TEOS/OMC at 1:1 and the addition of OMC and surfactant in the oil phase, which had smaller particle sizes (476.5 nm), higher yield (95.8%), and higher entrapment efficiency (61.09%). FTIR results and in vitro release profile demonstrated that OMC/SiO_2_ nanoparticles were successfully prepared. In vitro release profile confirmed that OMC and surfactant added in oil phase had the lowest released rate, which also suggested that OMC/SiO_2_ nanoparticles can delay OMC releasing.

In the future, we expect that OMC/SiO_2_ nanoparticles can be developed as a new type of sunscreen materials and could be applied in the cosmetics industry to achieve a safer and better UV-protective ability.

## Figures and Tables

**Table 1:** Change experimental parameters to the effect of octyl methoxy cinnamates (OMC)/SiO_2_ nanoparticles.

Sample	Agitating Speed (rpm)	pH Value	Surfactant	TEOS:OMC
1	600	1.5	Span 80	1:1
2	800	1.5	Span 80	1:1
3	1000	1.5	Span 80	1:1
4	1000	1.0	Span 80	1:1
5	1000	2.0	Span 80	1:1
6	1000	1.5	Span 80	1:1
7	1000	1.5	Span 80/Tween 20 = 1	1:1
8	1000	1.5	Span 80/Tween 20 = 1	2:3
9	1000	1.5	Span 80/Tween 20 = 1	1:2

**Table 2:** The effect of OMC and surfactant added in different phase.

Sample	Agitating Speed (rpm)	pH Value	Surfactant (Span 80/Tween 20)	Phase of OMC	Phase of Tween 20	TEOS: OMC
7	1000	1.5	1	water	oil	1:1
10	1000	1.5	1	water	water	1:1
11	1000	1.5	1	oil	oil	1:1

**Table 3:** Particle sizes and yield of OMC/SiO_2_ nanoparticles.

Sample	Yield (%)	Particle Sizes ± SD (nm)	PDI
1	33.65	1163.3 ± 107.8	0.56 ± 0.09
2	35.80	1147.2 ± 213.2	0.59 ± 0.04
3	79.90	1093.2 ± 95.1	0.44 ± 0.05
4	42.06	900.0 ± 200.7	0.65 ± 0.09
5	24.25	1517.7 ± 156.0	0.40 ± 0.03
6	79.90	1093.2 ± 200.7	0.44 ± 0.05
7	87.19	683.0 ± 52.1	0.30 ± 0.01
8	80.05	1022.4 ± 70.4	0.24 ± 0.19
9	64.51	1237.0 ± 116.0	0.13 ± 0.08
10	74.38	796.3 ± 33.2	0.42 ± 0.18
11	95.80	476.5 ± 06.9	0.18 ± 0.15
